# Rethinking Lymphadenectomy in Cutaneous Melanoma: From Routine Practice to Selective Indication: A Narrative Review

**DOI:** 10.3390/medicina61091722

**Published:** 2025-09-22

**Authors:** Matteo Matteucci, Antonio Pesce, Salvatore Guarino, Diletta Cassini, Bruno Cirillo, Carlo Boselli, Vito D’Andrea, Marco Artico, Flavio Forte, Piero Covarelli, Roberto Cirocchi

**Affiliations:** 1 Department of General Surgery, University of Milan, 20122 Milan, Italy; matteo.matteucci@unimi.it; 2 Department of Surgery, Azienda Unità Sanitaria Locale (AUSL) Ferrara, 44121 Ferrara, Italy; antonio.pesce@unife.it; 3 Department of General Surgery, Istituto di Ricovero e Cura a Carattere Scientifico (IRCCS) Multimedica Sesto San Giovanni, 20099 Sesto San Giovanni, Italy; salvatore.guarino@gmail.com; 4 ASST Nord Milano-Department of General and Rery, Sesto San Giovanni Hospital, 20099 Sesto San Giovanni, Italy; diletta.cassini@asst-nordmilano.it; 5 Department of Surgery, Sapienza University, 00161 Rome, Italy; bruno.cirillo@uniroma1.it (B.C.); vito.dandrea@uniroma1.it (V.D.); 6 Department of Medicine and Surgery, University of Perugia, 06129 Perugia, Italy; carlo.boselli@unipg.it (C.B.); piero.covarelli@unipg.it (P.C.); 7 Department of Sensory Organs, Sapienza University, 00161 Rome, Italy; marco.artico@uniroma1.it (M.A.); flavioforte@hotmail.com (F.F.)

**Keywords:** cutaneous melanoma, lymph node dissection, sentinel lymph node biopsy, narrative review, complete lymphadenectomy, elective lymph node dissection

## Abstract

*Background and Objectives*: Lymph node management in cutaneous melanoma has undergone a paradigm shift, transitioning from routine complete lymph node dissection (CLND) to a more selective, individualized approach. This narrative review explores the historical evolution, current evidence and clinical guidelines surrounding lymphadenectomy for a patient with Stage III of melanoma. *Materials and Methods*: A comprehensive literature search was conducted across PubMed, Scopus and Web of Science, focusing on randomized controlled trials, meta-analyses and updated international guidelines published in the past 15 years. *Results*: Traditional surgical approaches favored radical lymphadenectomy for regional disease control. However, pivotal trials such as the Multicenter Selective Lymphadenectomy Trial II (MSLT-II) and German Dermatologic Cooperative Oncology Group Selective Lymphadenectomy Trial (DeCOG-SLT) demonstrated no survival advantage from immediate CLND following a positive sentinel lymph node biopsy (SLNB), underscoring increased surgical morbidity. Consequently, guidelines from Associazione Italiana di Oncologia Medica (AIOM), the European Society for Medical Oncology (ESMO), and the National Comprehensive Cancer Network (NCCN) now endorse SLNB as the standard for nodal staging, reserving CLND for select high-risk cases. *Conclusions*: The role of lymphadenectomy in melanoma is increasingly becoming selective, shaped by tumor burden, nodal involvement and response to systemic therapy. SLNB remains central to staging and treatment planning, while CLND is no longer routine. Continued clinical trials and integration with immunotherapy will further refine surgical strategies in melanoma care.

## 1. Introduction

Cutaneous melanoma is an aggressive skin tumor originating from melanocytes, pigment-producing cells of the epidermis. Despite representing only about 1% of all skin cancers, it is responsible for the majority of skin cancer-related deaths, reflecting its highly invasive nature. Over the past decades, the incidence of melanoma has been steadily increasing worldwide. In 2020, approximately 325,000 new cases and 57,000 deaths were reported globally, with the highest age-standardized incidence observed in Australia and New Zealand, followed by North America and Northern Europe. In contrast, substantially lower rates are reported in Asia, Africa, and Latin America, a disparity largely explained by differences in skin phototype and in patterns of ultraviolet (UV) exposure [1,2,3]. In Italy, according to AIOM guidelines, cutaneous melanoma represents the second most frequent cancer in the male sex and the third most frequent tumor in the female sex [4]. UV radiation remains the primary environmental risk factor, accounting for more than 80% of cases, as it promotes oxidative stress and DNA damage in melanocytes, thereby favoring oncogenic transformation [5,6,7]. At the molecular level, melanoma pathogenesis is driven by genetic alterations in pathways that regulate proliferation, differentiation and apoptosis, with recurrent mutations in genes such as BRAF, NRAS, NF1, KIT, TERT, and CDKN2A [8,9].

Melanoma encompasses several subtypes, each associated with distinct etiological and molecular features. Superficial spreading melanoma is the most common form, especially in fair-skinned populations, and typically arises in intermittently sun-exposed areas, often harboring BRAF V600E mutations. Nodular melanoma, by contrast, is characterized by rapid vertical growth. This subtype is associated with an unfavorable prognosis because it tends to present at greater thickness. Acral lentiginous melanoma, which is more prevalent among individuals with darker skin types, typically occurs on glabrous sites such as the palms, soles, and nail beds. Unlike other melanoma subtypes, it is not strongly associated with UV exposure. Other less common variants, including mucosal and uveal melanomas, pose particular clinical challenges due to their aggressiveness and frequent progression to advanced stages [10,11,12,13].

Cutaneous melanoma is currently staged using the 8th edition of the AJCC staging system [14], based on tumor (T), nodes (N) and metastases (M) classification. The tumor category is based on Breslow tumor thickness and on the presence or absence of ulceration. Tumor mitotic rate (TMR) is no longer a T category criterion but recent studies suggest a reconsideration of TMR prognostic role due to its association with decreased overall survival (OS), melanoma specific survival (MSS) and recurrence-free survival (RFS) [15,16,17]. Node (N) category is based on lymph nodes metastasis and on non-nodal regional metastasis, such as in-transit, micro-satellite, satellite and subcutaneous metastasis. The extracapsular extension (ECE) is not considered in N category, but recent studies suggest that ECE status should be assessed due to its potential prognostic value [18,19,20,21]. M category, instead, designates distant metastases. According to T, N and M classification, melanomas can be grouped into clinical and pathological stages. While Stage 0 indicates a melanoma in situ curable with only surgical excision, Stages III and IV of the disease are, respectively, defined by its spread to lymph nodes and by distant metastases [14].

Staging defines prognosis and guides treatment, particularly decisions regarding sentinel lymph node biopsy, adjuvant systemic therapy, and clinical trial eligibility. In fact, the management of regional lymph nodes in patients with cutaneous melanoma has undergone a profound transformation over the past several decades. This evolution reflects significant advances in surgical techniques, diagnostic modalities and a growing understanding of melanoma biology and its metastatic behavior.

A critical aspect of melanoma progression is its propensity to spread via the lymphatic system. For this reason, sentinel lymph node biopsy (SLNB) has become a cornerstone of melanoma staging. Introduced in the 1990s, the procedure involves peritumoral injection of radiotracers and/or vital dyes to map lymphatic drainage and identify the sentinel lymph node (SLN) intraoperatively [22,23,24,25,26]. Current guidelines [4,27,28] recommend SLNB for patients with melanomas greater than 0.8 mm in Breslow thickness and selectively in those with thinner tumors that exhibit high-risk features such as ulceration or high mitotic index. While SLNB is not considered a therapeutic intervention in itself, its diagnostic and prognostic value is widely recognized. In fact, histopathological and immunohistochemical analysis of the SLN provides critical prognostic information, as nodal status is among the most powerful predictors of recurrence and survival in melanoma. A positive SLNB upstages the disease to Stage III, directly influencing treatment decisions. Historically, complete lymphadenectomy was considered the standard of care for patients presenting with clinically palpable lymphadenopathy or following the identification of a positive SLN. This approach was grounded in the oncologic principle that early removal of regional nodal metastases could potentially interrupt disease progression, thereby improving disease-specific survival and overall outcomes. However, this paradigm began to shift with the emergence of large-scale prospective randomized controlled trials, most notably the Multicenter Selective Lymphadenectomy Trial II (MSLT-II) [29] and the German Dermatologic Cooperative Oncology Group Selective Lymphadenectomy Trial (DeCOG-SLT trial) [30]. The accumulated evidence from these studies demonstrated that immediate completion lymph node dissection (CLND) did not confer a statistically significant advantage in melanoma-specific survival compared to observation alone, although it did improve regional disease control. Moreover, CLND was associated with a higher risk of surgical morbidity, including lymphedema, wound complications, and sensory neuropathies. These adverse effects can significantly impair patients’ quality of life [29,30]. These findings have prompted a major paradigm shift in clinical practice, favoring more individualized and risk-adapted strategies over routine and universal lymphadenectomy.

As a result, the current standard of care has shifted away from mandatory CLND for patients with positive SLNB. Careful nodal observation, coupled with adjuvant systemic therapies, has become the preferred strategy in many guidelines for patients with Stage III of disease [4,27,28]. Nevertheless, an important clinical gap remains. Questions persist regarding whether specific patient subgroups, such as those with a high nodal tumor burden, extranodal extension or biologically aggressive disease, might still derive therapeutic benefit from CLND. Additionally, while nodal observation is safe in most cases, it requires reliable imaging follow-up and patient compliance, which may not be universally available. This ongoing controversy underscores the need for more refined risk stratification tools, integrating histopathological features, molecular biomarkers and imaging techniques, to identify which patients may truly benefit from surgical escalation. Until such precision approaches are validated, the decision to perform CLND after a positive SLNB remains individualized, balancing oncologic risk, patient preferences and potential morbidity.

This narrative review aims to provide a comprehensive examination of the evolving role of lymphadenectomy in cutaneous melanoma, highlighting the shift from routine CLND to more selective strategies. By synthesizing the latest clinical trial evidence and current surgical principles, this review seeks to clarify ongoing controversies and to underscore the need for an updated appraisal of current literature to better define the contemporary management in melanoma patients with Stage III disease.

## 2. Materials and Methods

A comprehensive literature search was performed using three major biomedical databases: PubMed, Scopus, and Web of Science. No temporal limits were applied in the literature search in order to capture both historical and contemporary evidence on the management of regional lymph nodes in cutaneous melanoma. A narrative review methodology was chosen for this work in order to provide a comprehensive and contextualized overview: in fact, by synthesizing diverse strands of evidence and clinical reasoning, this approach provides a broad yet critical appraisal of the literature.

**Figure 1:**
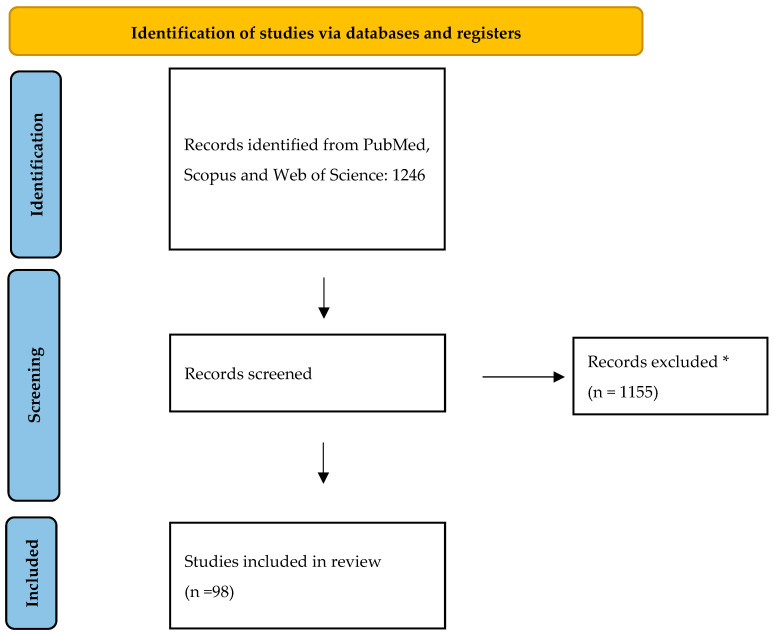
Flow diagram of identification of studies included in our review. * Records excluded because non-English language publications, case reports, editorials, or narrative commentaries without original data.

The strategy incorporated a targeted set of keywords, including “cutaneous melanoma AND elective lymph node dissection OR elective lymphadenectomy AND completion lymph node dissection OR completion lymphadenectomy AND therapeutic lymph node dissection AND sentinel lymph node biopsy”. The initial search across PubMed, Scopus, and Web of Science yielded a total of 1246 records. After removing duplicates, articles were screened based on titles and abstracts independently by two different authors (M.M. and R.C.). Inclusion criteria were randomized controlled trials, prospective or retrospective cohort studies, systematic reviews, meta-analyses and clinical practice guidelines regarding patients with only cutaneous melanoma, that constitute the highest tiers of evidence-based medicine, providing a robust framework for evaluating the safety, efficacy and optimal indications of SLNB and lymph node dissection. Exclusion criteria were non-English language publications, case reports, editorials, or narrative commentaries without original data and studies focusing exclusively on non-cutaneous melanoma subtypes (e.g., uveal or mucosal). 98 studies were ultimately used to prepare this narrative review. The searching strategy is reported in Figure 1.

No statistical analysis was performed.

## 3. From Handley to Morton: The Development of Lymphatic Surgery in Melanoma

A clear understanding of the developments in regional lymph node management requires familiarity with the specific terminology and definitions of the surgical techniques currently employed. The term “elective lymph node dissection” (ELND) refers to surgical removal of all lymph nodes in a regional basin without prior radiological or pathological evidence of nodal metastases. The term “therapeutic lymph node dissection” (TLND) is defined as surgical removal of all lymph nodes in a regional basin for clinically or macroscopic radiologically nodal metastases. Instead, by the term “Completion lymph node dissection” (CLND) we mean the surgical lymph node dissection after microscopic metastasis are identified, typically following a positive SLNB.

The origins of lymph node dissection in the treatment of cutaneous melanoma can be traced back to the early 20th century, rooted in the prevailing concept of regional disease control. Among the earliest and most influential figures in this domain was William Heneage Ogilvie Handley, a British surgeon and pathologist. In 1905, Handley published a landmark monograph in which he proposed a hypothesis on the lymphogenous spread of melanoma [31]. Through meticulous anatomical dissections and the innovative use of mercury injections into lymphatic vessels, Handley demonstrated a centrifugal pattern of lymphatic drainage from cutaneous melanomas to regional nodal basins. He theorized that tumor cells disseminated along these pathways, forming microscopic satellite metastases and eventually colonizing regional lymph nodes. Based on these observations, he advocated for wide local excision of the primary tumor in continuity with the draining lymphatic basin, even in the absence of clinically palpable adenopathy. This procedure, later termed elective lymph node dissection (ELND), was conceived as both a staging tool and a potentially curative intervention.

Throughout the early and mid-20th century, Handley’s principles were widely embraced by surgical oncologists [32,33,34,35]. ELND became increasingly standardized, particularly in patients with thicker primary melanomas or lesions located in anatomically high-risk regions such as the head and neck or extremities [36,37,38]. Surgeons like Herbert Snow [39] and Alexander Brunschwig [40] further advanced this surgical doctrine, promoting radical lymphadenectomy as a strategy to interrupt regional spread and improve survival outcomes. These extensive dissections reflected the aggressive surgical philosophy of the era.

Yet despite its widespread adoption, the clinical utility of ELND remained a subject of ongoing debate [41,42,43,44,45,46,47]. Early randomized trials, including those conducted by the Intergroup Melanoma Surgical Program [48] and the WHO Melanoma Group [49] demonstrated no significant improvement in overall survival for patients undergoing ELND compared with observation, although modest benefits in regional disease control were observed in selected subgroups, such as younger patients under 60 years of age, those with non-ulcerated tumors, with intermediate-thickness melanomas (1–2 mm) and with melanoma located at extremities.

The absence of randomized controlled data and the inability to reliably identify patients harboring occult nodal metastases raised concerns about overtreatment. Pathologic examination of dissected nodal basins revealed that a significant proportion of patients undergoing ELND had no histologic evidence of nodal involvement, suggesting that many were exposed to unnecessary surgical risk.

By the 1970s and 1980s, the landscape began to shift. Advances in histopathologic techniques and a more nuanced understanding of melanoma biology prompted clinicians and researchers to seek less invasive methods for staging the regional nodal basin, methods that could spare patients from the morbidity associated with extensive dissections. This search culminated in the development of the sentinel lymph node biopsy (SLNB) technique, which would prove to be a transformative milestone in melanoma surgery.

Pioneered by Donald L. Morton [50] and colleagues in 1992, SLNB was initially validated in melanoma and later extended to other solid tumors, including breast cancer. The technique was based on the principle of intraoperative lymphatic mapping, allowing surgeons to identify and analyze the initial lymph node(s) receiving drainage from the primary tumor [51,52,53,54,55]. SLNB offered a minimally invasive method for regional staging, preserving oncologic accuracy while significantly reducing the morbidity associated with ELND. Since Morton’s seminal study in 1992, numerous investigations have been conducted to validate the use of sentinel lymph node (SLN) biopsy in melanoma staging [56,57,58,59,60,61]. A meta-analysis [62] encompassing 71 of these studies, including over 25,000 patients, evaluated the reliability and accuracy of SLN biopsy as a staging procedure. The analysis reported a successful identification rate of 98.1% and a false-negative rate of 12.5%, confirming the procedure’s technical feasibility and reproducibility. Furthermore, Gerhenswald et al. [63] has demonstrated that SLN biopsy represents the single most important prognostic factor in patients with cutaneous melanoma without clinically evident metastases, providing critical information for risk stratification and therapeutic decision-making.

**Figure 2:**
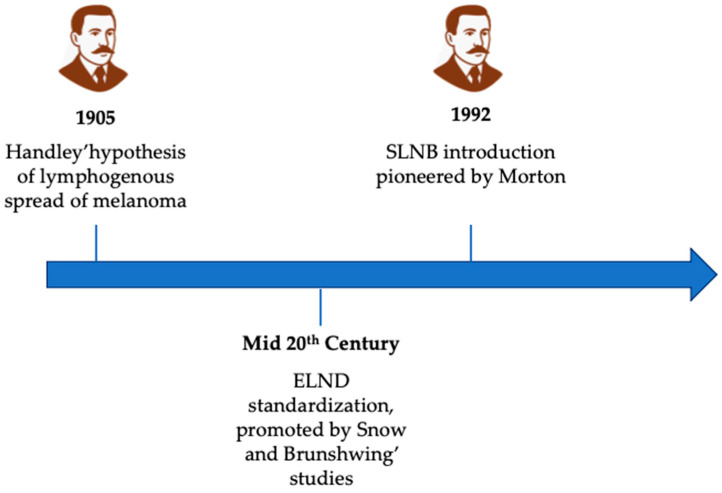
Timeline of lymph node dissection in the treatment of cutaneous melanoma. ELND: elective lymph node dissection; SLNB: sentinel lymph node biopsy.

The timeline of the history of lymph node dissection and SLNB is illustrated in Figure 2.

## 4. Sentinel Lymph Node Biopsy and Completion Lymph Node Dissection: Evidence from Clinical Trials

While the data have reinforced the clinical benefit of performing SLNB for its prognostic value, its role in guiding CLND has been more controversial and it has been rigorously evaluated through several studies. Historically, the sentinel lymph node was considered representative of the entire regional nodal basin. Consequently, a positive sentinel node was thought to indicate the need for completion lymph node dissection, with the aim of eradicating metastatic disease throughout the corresponding nodal basin. In the largest cohort of patients with a positive sentinel lymph node who underwent completion lymph node dissection, encompassing over 1500 individuals, non-sentinel lymph node (NSLN) status emerged as an independent prognostic factor for melanoma-specific survival [64]. Nevertheless, later studies demonstrated that only 12–20% [65] of patients actually had tumor-positive NSLNs at the time of dissection. Based on these studies, three large randomized controlled trials were conducted in order to assess the necessity of CLND after a positive SLNB.

One of the earliest and most influential investigations was the Multicenter Selective Lymphadenectomy Trial I (MSLT-I) [66]. This landmark study compared two approaches: wide excision plus SLNB (with immediate CLND if the SLNB was positive) versus wide excision followed by nodal observation, with delayed therapeutic lymphadenectomy in the event of nodal recurrence. Although SLNB did not demonstrate a statistically significant improvement in overall survival compared to nodal observation, it did yield a notable advantage in disease-free survival. Importantly, SLNB provided critical prognostic information, particularly for patients with intermediate-thickness melanomas (1.2–3.5 mm), by enabling early detection of micrometastases and guiding subsequent therapeutic decisions. The procedure also contributed to improved regional disease control.

Building on these findings, the MSLT-II trial [29] addressed a pivotal clinical question: should patients with a positive sentinel lymph node undergo immediate CLND, or can they be safely managed with active surveillance using regular nodal ultrasound? Enrolling over 1900 patients, the MSLT-II trial found no statistically significant difference in melanoma-specific survival between the immediate CLND group and the observation group. However, immediate CLND did reduce the risk of regional nodal recurrence and allowed for more accurate staging, identifying additional positive non-sentinel nodes in approximately 12% of patients. Despite these benefits, the procedure was associated with increased surgical morbidity, including lymphedema, wound infections, and nerve injuries [8].

These results were further supported by the DeCOG-SLT trial [30], which focused specifically on patients with micrometastatic involvement of the sentinel node. The trial confirmed that omitting CLND did not negatively impact distant metastasis-free survival or overall survival, reinforcing observation as a safe and effective strategy in appropriately selected patients. Moreover, the DeCOG-SLT trial highlighted the importance of individualized patient selection, particularly the assessment of tumor burden within the sentinel node, as a key factor in guiding management decisions [30].

Together, these trials have contributed to a more nuanced understanding of nodal management in melanoma, shifting the emphasis from routine completion dissection to selective, evidence-based strategies that prioritize both oncologic outcomes and patient quality of life.

## 5. Complications Associated with Lymphadenectomy

Completion lymph node dissection (CLND), while historically considered a standard approach for managing nodal metastases in melanoma, carries a significant burden of postoperative morbidity. These complications not only affect short-term recovery but also have lasting impacts on patient quality of life.

Among the most frequent and debilitating complications is lymphedema, which can persist chronically and severely impair limb function. Reported incidence ranges from 20% to 50%, influenced by factors such as the anatomical site of dissection, extent of nodal clearance, patient obesity, and the use of adjuvant radiation therapy [67,68]. Notably, inguinal lymphadenectomy is associated with a higher risk of lymphedema compared to axillary dissection, due to differences in lymphatic architecture and anatomical complexity [69]. Chronic lymphedema predisposes patients to recurrent cellulitis and functional limitations, making it one of the most feared long-term outcomes of CLND.

Wound complications, including infection, necrosis, and delayed healing, affect approximately 10–30% of patients undergoing CLND [7,70]. The Groin Lymphadenectomy in Melanoma (GOLM) study [71] identified several independent predictors of wound morbidity, such as high body mass index, smoking status, and extensive nodal dissection. These factors underscore the importance of preoperative risk stratification and perioperative optimization.

Seroma formation is another common early postoperative issue, occurring in 20–40% of cases. While often benign, seromas may require repeated aspirations and can increase the risk of secondary infection [70].

Nerve injury is a particularly concerning complication, especially in anatomically complex regions. In inguinal dissections, the femoral, obturator, and lateral femoral cutaneous nerves are vulnerable, while axillary dissections may affect the long thoracic and thoracodorsal nerves. These injuries can result in sensory deficits, neuropathic pain, and motor weakness, with long-term sequelae documented in up to 15% of patients [72].

The cumulative morbidity associated with CLND was a driving force behind the design of pivotal trials such as MSLT-II [29] and DeCOG-SLT [30]. These studies demonstrated that immediate CLND following a positive SLNB did not improve melanoma-specific survival but significantly increased complication rates. In the MSLT-II trial, the incidence of lymphedema was markedly higher in the CLND group (24.1%) compared to the observation arm (6.3%) [29]. Similarly, the DeCOG-SLT trial reported a 19% overall complication rate in patients undergoing CLND [30].

Complications associated with CLND and SLNB are resumed in Table 1.

## 6. Current Guidelines on Sentinel Lymph Node Biopsy and Lymphadenectomy in Melanoma: AIOM, ESMO, and NCCN Perspectives

Contemporary clinical guidelines from leading oncology societies, including the Italian Association of Medical Oncology (AIOM) [4], the European Society for Medical Oncology (ESMO) [27] and the National Comprehensive Cancer Network (NCCN) [28], reflect a unified shift in the management of regional lymph nodes in melanoma, emphasizing precision staging and minimizing unnecessary surgical morbidity.

All three organizations endorse sentinel lymph node biopsy (SLNB) as the standard of care for staging clinically node-negative patients with intermediate or high-risk primary cutaneous melanoma. Specifically, SLNB is recommended for tumors with a Breslow thickness ≥ 0.8 mm, or for thinner lesions exhibiting adverse features such as ulceration or high mitotic rate.

In contrast, elective lymph node dissection (ELND) is no longer routinely advised following a positive SLNB. This recommendation is grounded in robust evidence from randomized trials (e.g., MSLT-II [29], DeCOG-SLT [30]) demonstrating no melanoma-specific survival benefit and a higher rate of complications with immediate CLND. Instead, guidelines [4,27,28] advocate for active nodal surveillance, typically via high-resolution ultrasound, reserving CLND for patients with clinically evident nodal disease, radiologically confirmed metastases or high tumor burden within the sentinel node.

**Figure 3:**
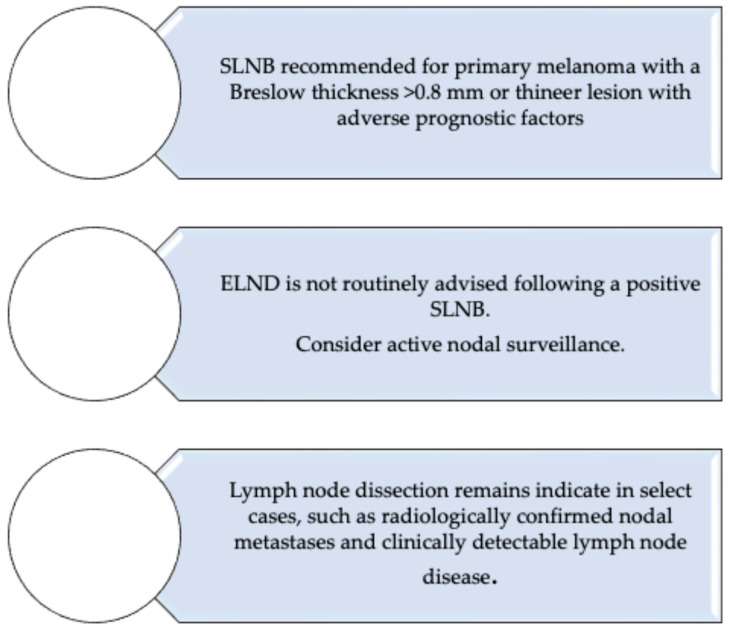
Flowchart of current guidelines on SLNB and lymph node dissection for cutaneous melanoma. SLNB: sentinel lymph node biopsy; ELND: elective lymph node dissection.

Collectively, these guidelines reflect a paradigm shift: lymph node surgery is no longer a default intervention but a selective tool, guided by tumor biology, imaging and systemic treatment strategies. A flowchart summarizing these recommendations is presented in Figure 3.

## 7. The Evolving Role of Lymph Node Dissection in the Era of Systemic Therapy for Melanoma

The advent of immune checkpoint inhibitors and targeted therapies has dramatically transformed the therapeutic landscape of Stage III cutaneous melanoma, reshaping the role of lymph node dissection within a broader, multidisciplinary framework [73,74,75,76,77,78].

Recent clinical trials have demonstrated that adjuvant systemic therapies significantly improve both relapse-free survival (RFS) and overall survival (OS) in patients with resected Stage III melanoma. The KEYNOTE-054 trial [79] showed that pembrolizumab reduced the risk of recurrence by 43% compared to placebo (hazard ratio 0.57; *p* < 0.001), with a 3-year RFS of 63.7% versus 44.1%. Similarly, the CheckMate-238 trial [80] reported superior RFS with nivolumab compared to ipilimumab (58% vs. 45% at 3 years; HR 0.65; *p* < 0.001). For patients harboring BRAF mutations, the COMBI-AD trial [81] demonstrated that the combination of dabrafenib and trametinib improved 3-year RFS to 58%, compared to 39% with placebo.

These findings underscore the critical importance of accurate nodal staging, primarily achieved through sentinel lymph node biopsy (SLNB), in identifying candidates for adjuvant systemic therapy. As a result, CLND is no longer routinely performed in SLN-positive patients, given the lack of survival benefit and the increased risk of surgical morbidity.

Emerging evidence from neoadjuvant trials further challenges the traditional indications for lymphadenectomy. The OpACIN-neo [82] and PRADO [83] trials investigated neoadjuvant immune checkpoint blockade in patients with Stage III melanoma. In the PRADO study, 61% of patients achieved a major pathologic response (defined as ≤10% viable tumor) following neoadjuvant treatment with nivolumab and ipilimumab. In these responders, therapeutic lymph node dissection was omitted, and no excess recurrences were observed at 2-year follow-up. These results suggest that pathologic response may serve as a reliable biomarker for surgical de-escalation, paving the way for more personalized and less invasive treatment strategies.

In light of these developments, the role of lymph node dissection has become increasingly selective and context-dependent. While CLND remains appropriate in cases of gross nodal disease, progression during systemic therapy, or symptomatic lymphadenopathy, it is no longer indicated as a default intervention following a positive SLNB. Instead, surgical management is now tailored to complement systemic approaches, reflecting a paradigm shift toward precision oncology in melanoma care [84].

## 8. The Limits of Sentinel Node Prognostic Value and Imaging-Based Surveillance in Melanoma Care

Despite significant advances in the management of cutaneous melanoma, substantial controversy persists regarding the optimal treatment of patients with nodal disease. Notably, the pivotal adjuvant immunotherapy trials that established immune checkpoint inhibitors and targeted therapies as standards of care were conducted in the CLND era, when all patients with a positive sentinel node typically underwent nodal dissection. As a result, trial populations had more complete pathological staging and potentially different disease biology compared to current patients, many of whom retain their nodal basins under observation after a positive SLNB. This discrepancy raises important questions regarding the applicability of existing survival and recurrence data to present-day practice, where adjuvant therapies are increasingly administered without full pathological nodal clearance.

Emerging evidence indicates that the prognostic significance of sentinel lymph node (SLN) status in cutaneous melanoma varies significantly depending on the primary tumor’s anatomical site. For melanomas located in the head and neck (HN) region, SLN positivity is strongly associated with worse survival outcomes: one study involving 331 patients reported a 5-year overall survival of 91.2% in SLN-negative cases versus only 48.7% in SLN-positive cases, with SLN status being the most powerful predictor of recurrence (hazard ratio ≈ 20.6, *p* < 0.0001) [85]. A meta-analysis confirmed that SLN positivity nearly triples the risk of poor prognosis in HN melanoma (HR = 3.42; 95% CI 1.94–6.02; *p* < 0.001), while SLN biopsy itself was associated with a moderate improvement in overall survival (HR = 0.845; 95% CI 0.725–0.986; *p* = 0.032) [86]. Importantly, scalp melanomas appear to fare particularly poorly: SLN positivity was identified as the strongest predictor of overall survival in scalp melanoma, whereas in non-scalp HN melanomas, tumor thickness and ulceration had greater prognostic impact. Finally, anatomical complexity in lymphatic drainage in the HN region leads to lower SLN positivity rates (~12.9%) but higher false-negative rates and regional recurrence compared to other sites [87].

High-resolution ultrasonography significantly outperforms computed tomography (CT) in the surveillance of regional lymph nodes for melanoma patients. Meta-analytic data indicate that ultrasound achieves a markedly higher sensitivity (96%; 95% CrI: 85–99%) and specificity (99%; 95% CrI: 95–100%), compared to CT’s more modest sensitivity of 61% (95% CrI: 15–93%) and specificity of 97% (95% CrI: 70–100%) [88]. This superior diagnostic performance has been recognized in expert guidelines, which highlight that reliance on CT-alone surveillance risks delayed detection or missed lymph node metastases [89]. Moreover, in a large prospective study, ultrasonography combined with clinical examination demonstrated significantly higher sensitivity (0.94 vs. much lower for palpation) and specificity (0.98), underscoring its added value over physical exam alone [90,91]. Importantly, subgroup analyses from MSLT-II [29] data reveal that nearly half of nodal recurrences were identified by ultrasound alone, a proportion that rises to 65% in individuals with obesity, though no associated improvement in melanoma-specific survival was detected [89]. Despite its high sensitivity and specificity, ultrasound-based nodal surveillance has several important limitations. First, the diagnostic accuracy of ultrasonography is highly operator-dependent, with substantial variability between institutions and examiners, particularly in challenging anatomical regions such as the head and neck. Second, ultrasonography is best suited for superficial nodal basins and may have reduced sensitivity for deeply located or anatomically less accessible nodes, where CT or PET/CT may still provide complementary information. Third, while ultrasound can reliably detect nodal metastases at an earlier and often subclinical stage, there is limited evidence that this earlier detection translates into improved melanoma-specific survival, as highlighted by subgroup analyses of the MSLT-II trial [29]. In addition, ultrasound requires repeated examinations at relatively short intervals, raising issues of patient compliance, resource utilization, and costs in long-term follow-up. Conversely, CT offers standardized cross-sectional imaging and can simultaneously evaluate visceral sites of recurrence, but it is less sensitive for small nodal metastases, exposes patients to ionizing radiation, and has lower cost-effectiveness when used routinely for nodal surveillance.

## 9. From Completion Lymphadenectomy to Index Node Resection: Evolving Surgical Strategies and the MSLT-3 Trial

In recent years, the concept of index lymph node dissection (ILND), also referred to as “index node” management, has gained traction as a potentially less invasive alternative to completion lymph node dissection (CLND). This approach involves targeted removal of the single lymph node first identified by neoadjuvant immunotherapy, rather than performing extensive nodal dissection across all draining basins. In a multi-center analysis of patients treated with neoadjuvant ipilimumab–nivolumab, ILN response was highly representative of the entire basin: concordance with the TLND specimen was 99% at the patient level and 96% when compared against every individual node, supporting the ILN as a reliable surrogate for basin-wide response assessment. Building on this, the phase II PRADO extension (n = 99) operationalized ILN-guided personalization: patients achieving a major pathologic response (MPR; ≤10% viable tumor) in the ILN omitted TLND (59/60 did so) and adjuvant therapy, with markedly lower surgical morbidity and excellent 24-month outcomes (relapse-free survival 93%; distant metastasis-free survival 98%); partial responders had TLND only, and non-responders received TLND plus adjuvant therapy ± radiotherapy. Overall pathologic response was 72%, including 61% MPR, with grade 3–4 toxicity in 22% during the first 12 weeks [83]. These data underpin the ongoing randomized MSLT-3 trial, which is designed to test whether selective ILN resection after ~6 weeks of neoadjuvant immunotherapy is non-inferior to standard TLND for 2-year relapse-free survival in patients with macroscopic stage IIIB–D disease who achieve MPR; as of its latest public listing, the study is planned and coordinated through international cooperative groups [92]. Complementary observational work also emphasizes that SLNB and nodal surgery deliver durable regional control in most patients, providing clinical context for how response-adapted strategies might preserve oncologic safety while minimizing morbidity.

This evolving approach has been further reinforced by recent phase III trials. In the S1801 trial [93] (neoadjuvant + adjuvant vs. adjuvant-only pembrolizumab in resectable Stage IIIB–IV melanoma), patients receiving pembrolizumab before and after surgery had a significantly longer event-free survival (EFS): at a median follow-up of 14.7 months, the 2-year EFS was 72% (95% CI 64–80) in the neoadjuvant-adjuvant arm versus 49% (95% CI 41–59) in the adjuvant-only arm; the hazard ratio was 0.58, corresponding to a 42% reduction in event rate. The NADINA trial [94] (phase III, neoadjuvant ipilimumab + nivolumab vs. standard adjuvant nivolumab in macroscopic, resectable Stage III melanoma) similarly showed that neoadjuvant therapy improved outcomes. At 12 months, EFS was 83.7% in the neoadjuvant arm versus 57.2% in the adjuvant-only arm. Additionally, a major pathological response (MPR) was observed in 59% of patients in the neoadjuvant arm.

These data reinforce that earlier systemic therapy combined with response-adapted surgical strategies (like ILND or omitting extensive dissection in patients with strong pathologic response) may achieve comparable oncologic safety while reducing morbidity.

## 10. Melanin Pigmentation in Cutaneous Melanoma: Implications for Disease Progression, Lymph Node Involvement and Therapeutic Decision-Making

In cutaneous melanoma, melanin pigmentation is not merely a phenotypic trait but a biologically active factor that can modulate tumor behavior, therapeutic responsiveness, and the risk of lymphatic dissemination. Melanin’s dual role as a protectant against ultraviolet (UV) damage and as a modulator of tumor behavior necessitates rigorous assessment of pigmentation in clinical evaluations [7]. First, genetic variants of the melanocortin-1 receptor (MC1R), which affect pigmentation traits, significantly increase melanoma risk, both independently of skin phototype and in combination with other risk alleles (e.g., CDKN2A mutations) [95]. Numerous studies have highlighted that amelanotic or hypopigmented melanomas are often associated with more aggressive biological features, including higher mitotic rate, deeper invasion and a greater propensity for early lymph node involvement compared to their pigmented counterparts [96,97]. The degree of tumor pigmentation may therefore influence the indication for SLNB and even CLND.

Some analyses suggest that amelanotic melanomas, despite often being diagnosed at later stages due to their subtle clinical presentation, show a disproportionately higher rate of positive sentinel nodes relative to thickness-matched pigmented melanomas, warranting more aggressive nodal evaluation [96,97].

Pigmentation also affects treatment planning and expected responsiveness. Melanin has been shown to interfere with the efficacy of radiotherapy by scavenging reactive oxygen species, thus protecting tumor cells from radiation-induced damage [98]. Moreover, melanin biosynthesis is metabolically linked to immune evasion [7]. Pigmented melanoma cells may alter the tumor microenvironment through oxidative stress modulation and immunosuppressive metabolite release, potentially affecting the efficacy of immune checkpoint inhibitors (ICIs).

These findings support the integration of pigmentation assessment into routine melanoma staging and treatment planning. Parameters such as tumor pigmentation degree, presence of amelanotic features, MC1R genotype and phototype should be documented, as they can modify the urgency and scope of nodal staging procedures, the choice of adjuvant therapy and even the prognostic expectations in terms of metastatic risk and treatment response.

## 11. Conclusions

The management of regional lymph nodes in cutaneous melanoma has shifted dramatically over recent decades. Completion lymph node dissection (CLND), once routinely performed following sentinel lymph node biopsy (SLNB), is now reserved for selected high-risk cases, reflecting robust evidence from randomized trials, real-world studies, and the impact of systemic therapies on regional disease control. Current international guidelines [4,27,28] endorse SLNB as the primary staging tool for patients at intermediate or high risk, while routine CLND after a positive SLN is no longer recommended; active nodal surveillance is now preferred in most cases. The advent of adjuvant and neoadjuvant therapies further supports de-escalation of surgery, particularly in patients demonstrating favorable biological responses. This review synthesizes contemporary evidence on lymphadenectomy, highlighting the changing indications, prognostic implications of SLN status, and emerging strategies for personalized, risk-adapted nodal management. Table 2 summarizes the key studies included in our review, with their key findings and conclusions.

## Figures and Tables

**Table 1:** Complications associated with CLND and SLNB resumed from literature review.

Outcomes	SLNB	CLND	Comments
Surgical Morbidity	Low	Moderate-High	None
Lymphedema	3–7%	20–30%	Risk higher after CLND, particularly in inguinal region (MSLT-II, DeCOG-SLT).
Seroma Formation	1–5%	10–20%	None
Wound Infection	<5%	5–15%	None
Nerve Injuries	Rare	5–10%	None
Hospital Stay	1 day	1–3 days	CLND often requires longer postoperative monitoring.

**Table 2:** Key clinical trials and guidelines shaping nodal management in cutaneous melanoma, highlighting the shift from routine CLND to SLNB with surveillance and the integration of adjuvant and neoadjuvant systemic therapies.

Trial/Guideline	Population/Scope	Intervention/Focus	Key Findings/Recommendations
MSLT-II (2017) [29]	1900 patients with positive SLNB	CLND vs. nodal observation with ultrasound	CLND improved regional disease control but showed no melanoma-specific survival benefit compared to observation.
DeCOG-SLT (2016, 2019 final) [30]	Patients with positive SLNB	CLND vs. observation	No survival advantage for CLND; reinforced shift towards active surveillance after SLNB positivity.
COMBI-AD (2017) [81]	BRAF V600-mutant resected Stage III melanoma	Adjuvant dabrafenib + Trametinib Vs. Placebo	3-year RFS 58% Vs. 39% with placebo; established targeted therapy as standard adjuvant option in BRAF + patients.
OpACIN-neo (2019, 2023 update) [82]	Stage III melanoma	Neoadjuvant ipilimumab nivolumab (different dosing arms)	High pathologic response rates; responders had markedly improved RFS, supporting neoadjuvant immunotherapy as feasible and effective.
PRADO (2022) [83]	Stage III melanoma (extension of OpACIN-neo)	Neoadjuvant ipilimumab + nivolumab; response-directed surgery	61% major pathologic response; in responders, TLND was safely omitted without increased recurrence, supporting response-adapted surgery.
AIOM, ESMO, NCCN Guidelines [4,27,28]	Clinical practice recommendations	SLNB, CLND, surveillance, adjuvant and neoadjuvant therapy	All endorse SLNB for staging. CLND no longer routine after positive SLN (observation with ultrasound preferred). Adjuvant: anti–PD-1 or BRAF/MEK targeted therapy. Neoadjuvant: ICI in resectable Stage III, with potential for surgery de-escalation in responders.

## Data Availability

The data used to support the finding of this study are included within the article.
